# Digitally assisted learning in cardiac electrophysiology and cardiac implantable electronic devices: a Scientific Statement of the European Heart Rhythm Association of the ESC

**DOI:** 10.1093/europace/euag081

**Published:** 2026-04-13

**Authors:** Sabine Ernst, Alexandre Almorad, Patrick Badertscher, David Duncker, Anthony G Gallagher, Anthony Li, Ashley Nisbet, Roberto De Ponti, Andreu Porta-Sánchez, Lluis Mont, Marta De Riva, Andrea Sarkozy, Nili Schamroth, Emma Svennberg, Fleur Tjong, Serge A Trines, Laura Vitali-Serdoz, Serge Boveda

**Affiliations:** Department of Cardiology, Royal Brompton and Harefield NHS Foundation Trust/National Heart and Lung Institute, Imperial College London, SW3 6NP London, UK; Department of Cardiology, Heart Rhythm Management Center, CHU St Pierre, 1000 Brussels, Belgium; Department of Cardiology, University Hospital Basel, 4031 Basel, Switzerland; Department of Cardiology and Angiology, Hannover Heart Rhythm Center, Hannover Medical School, 30625 Hannover, Germany; Faculty of Life and Health Sciences, School of Medicine, Ulster University, BT15 1AP Belfast, UK; Department of Cardiology, St George's University Hospitals NHS Foundation Trust, SW1 7 0QT London, UK; Bristol Heart Institute, Bristol Royal Infirmary, BS2 8HW Bristol, UK; Department of Medicine and Surgery, University of Insubria, 21100 Varese, Italy; Arrhythmia Unit, Department of Cardiology, Hospital Clínic de Barcelona, 08036 Barcelona, Catalonia, Spain; Arrhythmia Unit, Department of Cardiology, Hospital Clínic de Barcelona, 08036 Barcelona, Catalonia, Spain; Department of Cardiology, Leiden University Medical Center, 2333 ZA Leiden, The Netherlands; Heart Rhythm Management Centre, Universitair Ziekenhuis Brussel, Vrije Universiteit Brussel, 1090 Jette, Belgium; Department of Cardiology, Rabin Medamical Center, 4941492 Petah Tikva, Israel; Department of Cardiology, Karolinska Institutet, Karolinska University Hospital Huddinge, SE-141 86 Stockholm, Sweden; Department of Cardiology, Heart Center, Amsterdam UMC, University of Amsterdam, 1105 AZ Amsterdam, The Netherlands; Department of Cardiology, Heart Lung Center, Leiden University Medical Center, 2333 ZA Leiden, The Netherlands; Arrhythmia and Electrophysiology Division, Department of Heart and Lung, Klinikum Fuerth, 90766 Fuerth, Germany; Cardiology—Heart Rhythm Management Department, Clinique Pasteur, 45 Avenue de Lombez, 31076 Toulouse, France

**Keywords:** Simulation Training, Digital Training, Cardiac electrophysiology, Proficiency-based progression, Catheter ablation, Cardiac implantable electronic devices

## Abstract

The rapid expansion of interventional electrophysiology (EP) and cardiac implantable electronic device (CIED) therapies has outpaced the capacity of traditional apprenticeship-based training to deliver standardized, equitable, and patient-safe skill acquisition across Europe. This scientific statement of European Heart Rhythm Association (EHRA) of the European Society of Cardiology (ESC) synthesizes the current landscape of digitally assisted learning for EP/CIED, provides a structured framework for implementation, and defines the responsibilities of scientific societies in establishing trustworthy, competency-based pathways. The document first reviews the technical principles and available digital modalities, emphasizing alignment of tool selection with learning objectives and staged progression. It then contrasts these capabilities with real-world uptake, highlighting fragmented access and strong reliance on industry-mediated training, alongside the emergence of neutral access models such as the EHRA Simulation Village. A central suggestion is the adoption of proficiency-based progression supported by protocolized evaluation: predefined and validated performance metrics, benchmarking against expert standards, deliberate practice with structured feedback, and summative assessment prior to patient procedures. Key implementation challenges include resource disparities and geographic inequities, limits in simulation fidelity for team-based and contextual decision-making, governance of industry relationships, and the need for protected educational time. Pragmatic solutions include tiered certification tracks, hybrid models combining digital and supervised clinical training, regional simulation hubs, minimum simulator specifications, ongoing curriculum updates, and coordinated stakeholder collaboration. Priority research needs are defined around clinical outcome validation, optimal curriculum design, technology development, and implementation science.

## Graphical Abstract

**Graphical Abstract:**
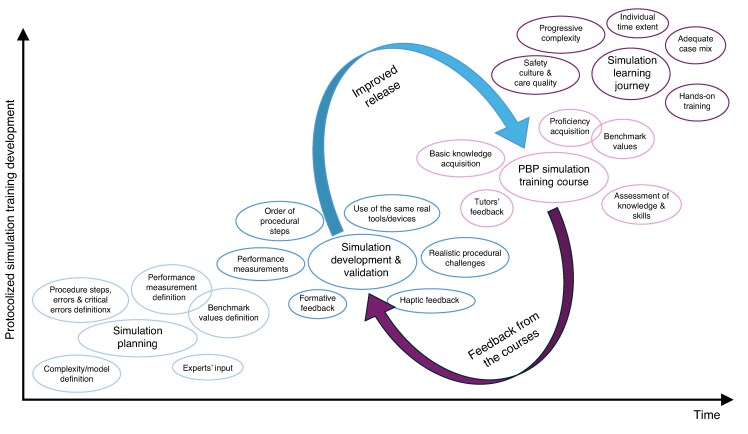

## Table of contents

1. Introduction
2. An overview of technical principles and existing systems
 2.1 Digitally assisted learning: definition and principles
 2.2 Benchtop phantoms and 3D-printed models
 2.3 High-fidelity haptic simulators
 2.4 Software-based modules
 2.5 Virtual and augmented reality
 2.6 Integration considerations
3. Where we are, where we go: current and future use of simulation
 3.1 Current landscape
  3.1.1 Academic and local initiatives
  3.1.2 Industry-driven training
  3.1.3 The EHRA simulation village: a neutral access model
 3.2 Future directions
4. The need for protocolized evaluation: the proficiencybased progression-moving from access to excellence
 4.1 Standards for simulation development and validation
 4.2 Building a proficiency-based training curriculum
 4.3 Integrating PBP into the learning journey
5. The need to invent new teaching methods
 5.1 The halstedian legacy’s end
 5.2 Digital era educational demands
 5.3 Four pillars for educational transformation
 5.4 Integration with existing frameworks
6. Educational pathways: advantages and difficulties moving toward certification
 6.1 Current certification framework: from theory to practice
 6.2 Integrating digital tools into certification pathways
 6.3 Advantages of digitally-enhanced certification
 6.4 Implementation challenges
 6.5 Pathways forward
7. Role of scientific societies
 7.1 Unique position and responsibilities
 7.2 Setting educational standards
 7.3 Facilitating evidence generation
 7.4 Ensuring global equity
 7.5 Neutral governance and stakeholder coordination
 7.6 Implementation strategies
8. Limitations and barriers
 8.1 Technical limitations
  8.1.1 Procedural complexity
  8.1.2 Haptic fidelity
 8.2 Educational barriers
  8.2.1 Skill transfer validation
  8.2.2 Systemic challenges
 8.3 Path forward despite limitations
9. Areas for research
 9.1 Priority research areas
10. Conclusion
Supplementary material
Acknowledgements
Data availability
References

What’s New?

- **Identifying a major implementation gap** in Europe: simulator access and formal curriculum integration remain limited despite high perceived usefulness.
- **Documenting heavy reliance on industry-mediated training**, raising concerns about educational neutrality, uneven access, and separation from structured assessment.
- **Proposing a society-governed framework** to integrate digitally assisted learning into EP/CIED training with defined responsibilities, minimum standards, and quality assurance.
- **Centres certification pathways on proficiency-based progression (PBP)** with protocolized evaluation
- **Outlining pragmatic implementation solutions** (tiered tracks, hybrid models, regional simulation hubs) and prioritizing research needs around outcome validation and implementation science.

## 1. Introduction

The safe and effective adoption of innovations in medical practice requires comprehensive theoretical and practical training for healthcare professionals. Digitally assisted simulator-based training has been successfully implemented in other high-stakes fields, most notably aviation, where it has been mandatory for pilot training for decades, and laparoscopic surgery, where simulation is now integral to surgical training curricula, with both demonstrating improved safety outcomes.^1,2^ Yet across Europe, a significant gap exists in coherent, standardized, and high-quality training programmes, particularly in interventional electrophysiology (EP) and cardiac implantable electronic devices (CIEDs) fields experiencing unprecedented scientific and technical advancement. The challenge is threefold: traditional training methods struggle with modern complexity, digital solutions remain fragmented, and implementation lacks academic oversight (see Supplementary material online, *Figure S1*).

Traditional EP and CIED fellowship training, while essential, faces mounting challenges including laboratory overcrowding, limited exposure to rare procedures, growing medico-legal exposure associated with complications during the early learning curve, and the ethical concerns of practicing on patients during the early learning curve. These limitations are compounded by significant disparities across European training environments, where infrastructure, device adoption, and digital integration vary widely between centres and countries.^3^

To date, EP simulation has not been systematically embedded within formal, competency-based academic training pathways across Europe.

The European Heart Rhythm Association (EHRA) of ESC consensus document on digital assisted learning addresses this critical need by (1) providing a comprehensive overview of available digital learning systems; (2) examining current implementation revealing a stark disconnection between need and availability; (3) establishing frameworks for PBP and objective assessment; (4) proposing innovative teaching methodologies; (5) outlining pathways towards formal certification; (6) defining the role of scientific societies; and (7) identifying barriers and research priorities. Our goal is to guide the integration of digitally-assisted learning into EP and CIED education, ensuring these powerful tools enhance rather than replace traditional training, ultimately improving patient care across Europe.

## 2. An overview of technical principles and existing systems

### 2.1 Digitally assisted learning: definition and principles

Building on the need for digital solutions outlined above, this section examines how digital tools encompass technologies to enhance medical education. In EP and CIED contexts, these tools serve dual purposes: delivering theoretical knowledge through structured online courses and advancing practical skills through simulation-based learning.

Healthcare simulation creates controlled environments that replicate real clinical events for practice, learning, evaluation, and systems understanding.^4^ Evidence demonstrates that simulator-based training reduces procedure times, radiation exposure, and supervisor dependence while improving performance, for example, in catheter placement and transseptal puncture.^5–8^

Effective EP and CIED simulators incorporate four key principles: realistic anatomical and physiological representation with haptic feedback; accurate replication of procedural steps; standardized, repeatable scenarios for proficiency development; and integrated assessment tools with structured feedback supporting competency-based progression.^4,9^

Ex vivo and live animal models have historically supported EP and CIED procedure training, contributing to early testing of mapping systems, catheters, pacing leads, and delivery systems (*Table 1*).^10^ They offer tactile realism, circulatory dynamics, respiratory motion, true ablation lesion generation, and uniquely permit direct pathological visualization following sacrifice. However, multiple factors now compel transition towards digital alternatives: ethical concerns regarding animal welfare; substantial acquisition and maintenance costs; complex logistics requiring dedicated facilities, veterinary oversight, and anaesthesia support; limited reproducibility across sessions; regulatory burden; biohazard management; anatomical differences limiting extrapolation to human procedures; and variable compatibility with contemporary devices optimized for human use. Digital training should therefore progressively replace or complement animal models.

**Table 1:** A non-exhaustive list of different simulators available classified by type

Category	Simulator Name	Manufacturer/Developer	Key Features	Supported Procedures
Physical phantoms and 3D-printed models				
	Heartroid	Heartroid/JMC	Rigid/semi-compliant cardiac chambers; accepts standard sheaths and catheters; and real contact force sensing	Vascular access, catheter navigation, transseptal puncture, and lead placement
	David Simulator	Mouzee	Electrogram generation; X-ray compatible; respiratory motion; and ablation capability	Vascular access, navigation, transseptal puncture, and ablation
	S-ICD Dummies	Boston Scientfic	S-ICD implantation	S-ICD implantation
High-fidelity haptic simulators				
	ANGIO Mentor EP	3D Systems (Littleton, CO, USA)/Simbionix	Force-feedback catheter interface; Simulated fluoroscopy; ICE integration; complete EP workflow	AF ablation, atrial flutter, SVT, VT ablation, and complication management
	Mentice VIST	Mentice (Gothenburg, Sweden)	Force-feedback motors; integrated fluoroscopy; electrogram simulation; CIED modules	EP procedures, venous access, CS cannulation, CSP, lead deployment, and leadless pacing
Software-based modules				
Browser-based	EP-Simulator	Various developers	Multi-module platform; real case simulations; expert-guided training	Pacing maneuvers, signal reading, ECG interpretation, and arrhythmia mechanisms
Browser-based	Epicardio Simulation	Epicardio	Multi-module platform; real case simulations; expert-guided training	Pacing maneuvers, ECG interpretation, and arrhythmia mechanisms
CIED training	Manufacturer-specific modules	Medtronic, Abbott, Biotronik, Boston Scientific	Device-specific programming; Troubleshooting; brand-specific features	Device programming, CRT optimization, and remote monitoring setup
Mapping simulation	CARTO Simulator	Biosense Webster (J&J)	Mapping, EGM interpretation, lesion metrics, and case review	Arrythmia mapping and ablation
Mapping simulation	EnSite Review Mode	Abbott	Mapping, EGM interpretation, lesion metrics, and case review	Arrythmia mapping and ablation
Mapping simulation	Opal Simulation	Boston Scientific	Mapping, EGM interpretation, Lesion metrics, and case review	Arrythmia mapping and ablation
Mapping simulation	Affera	Medtronic	Mapping, EGM interpretation, Lesion metrics, and case review	Arrythmia mapping and ablation
Mapping simulation	Colombus	Microport	Mapping, EGM interpretation, Lesion metrics, and case review	Arrythmia mapping and ablation
Virtual and augmented reality				
	CommandEP	Case Western Reserve University (Cleveland, OH, USA)	3D interactive anatomy; tracked controllers; spatial reasoning enhancement	Catheter orientation, ablation planning, and anatomical understanding
	Heart Rhythm VR	Heart Rhythm Society	S-ICD implantation module; pericardiocentesis module; Transcatheter PM in development	S-ICD implant, emergency procedures, and device implantation
	AVEIR simulator	Abbott	Leadless pacemaker implantation with haptic feedback	Leadless pacemaker implantation
Robotic navigation training				
	Stereotaxis Training Simulator	Stereotaxis	Web-based platform; browser-compatible; robotic navigation training	Robotic catheter manipulation, remote navigation, and magnetic navigation
Epicardial access training				
Epicardial simulator	EpiCardio Simulator	EpiCardio Solutions	Pericardial access training; fluoroscopy-compatible; realistic tissue layers	Epicardial access, subxiphoid puncture, epicardial mapping, and ablation
Hybrid and other systems				
Hybrid platform	Visible heart models	University of Minnesota	Reanimated hearts; direct visualization; educational platform	Anatomy education, procedure planning, and research applications
Minimal invasive surgery (MIS)	CADets		Epicardial access	Hybrid arryhtlmias procedures

### 2.2 Benchtop phantoms and 3D-printed models

**Figure 1:**
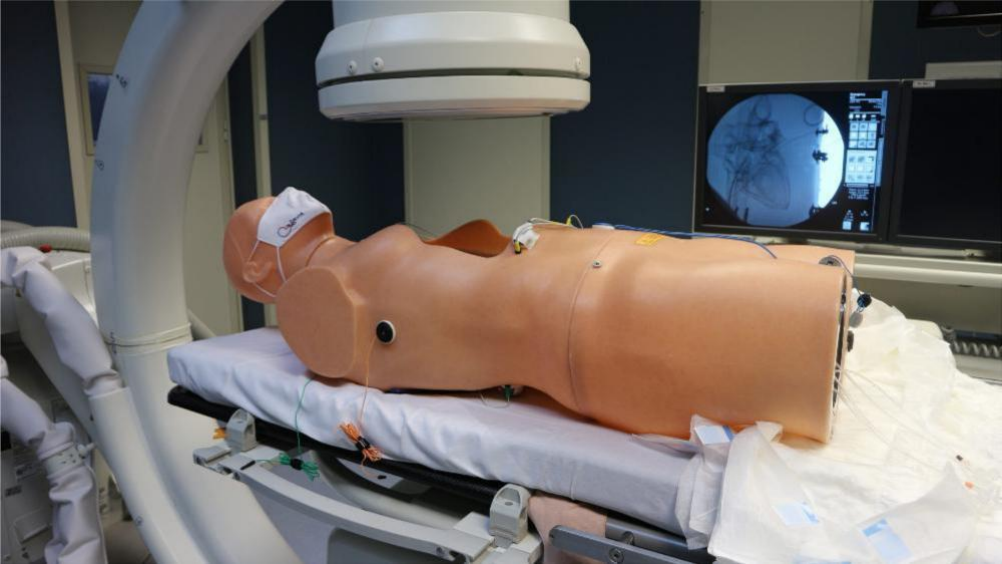
EP-David^TM^ simulator from mouzee (Turin, Italy).

Physical phantoms like the Heartroid™, EP-David™, and SINI Inc.™ simulators replicate cardiac anatomy and vascular pathways, accommodating standard sheaths, catheters, and pacing leads (*Figure 1*). These systems enable practice of vascular access, catheter navigation, transseptal puncture, and lead placement with real contact force-sensing catheters, providing authentic haptic feedback. The David system notably includes electrogram generation, X-ray compatibility, respiratory motion, and ablation capability. While cost-effective and reproducible, most lack full integration with advanced imaging modalities. Patient-specific 3D-printed models derived from CT or MRI allow case-specific rehearsal for complex anatomies, particularly valuable for congenital heart disease ablation and left bundle branch pacing procedures, when it comes to understanding lead-tissue interaction and specific septal anatomy.^11,12^

### 2.3 High-fidelity haptic simulators

**Figure 2:**
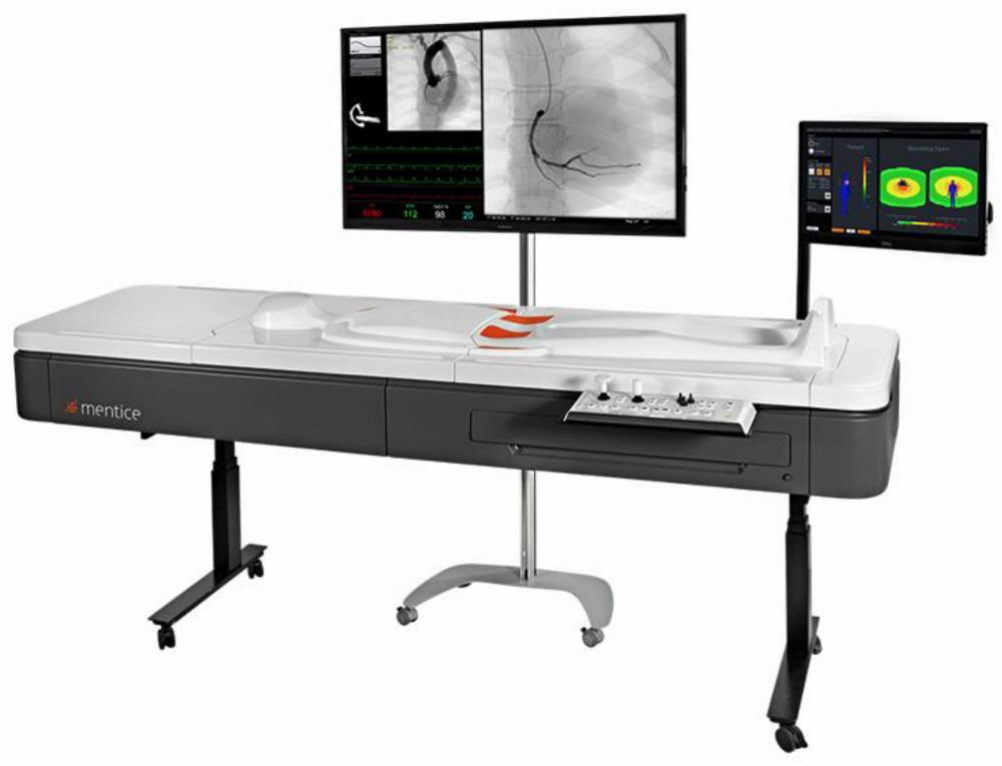
Mentice^TM^ simulator form surgical science with an angiogram of the coronary sinus taken from the CRT training module (Gothenburg, Sweden).

Commercial platforms such as ANGIO Mentor EP™ (3D Systems, Littleton, CO) and Mentice VIST™ (Mentice, Gothenburg, Sweden) employ proprietary catheter interfaces with force-feedback motors approximating contact force (*Figure 2*). These systems integrate simulated fluoroscopy, intracardiac echocardiography, hemodynamic signals, and electrograms, enabling complete procedural workflows for various arrhythmias and complication management. CIED-specific modules cover venous access, coronary sinus cannulation, lead deployment, including conduction system pacing (CSP), and leadless pacing. While highly immersive and validated for skill acquisition, limitations include substantial costs and tactile feedback that may not fully replicate clinical reality.^5,6,13^

A specific current limitation is the restricted number of anatomical variants available in most simulators (typically fewer than 10). However, since these systems are based on adaptable 3D models, libraries of anatomical variations can be expanded to address specific training needs, including complex or rare configurations. In this sense, simulators are scalable, integrating new scenarios, more specific or complex anatomies, and new technologies over time. On the other hand, tactile feedback has improved substantially, perfect replication of clinical reality remains inherently limited by constraints of a physics-based artificial simulation of human anatomy. Nevertheless, the objective is not absolute replication, but sufficient realism to enable skill acquisition and, critically, to build operator confidence. Trainees who have rehearsed procedures in simulated environments approach their initial clinical cases with greater self-assurance, reduced anxiety, and improved readiness, factors that independently contribute to procedural safety.

### 2.4 Software-based modules

**Figure 3:**
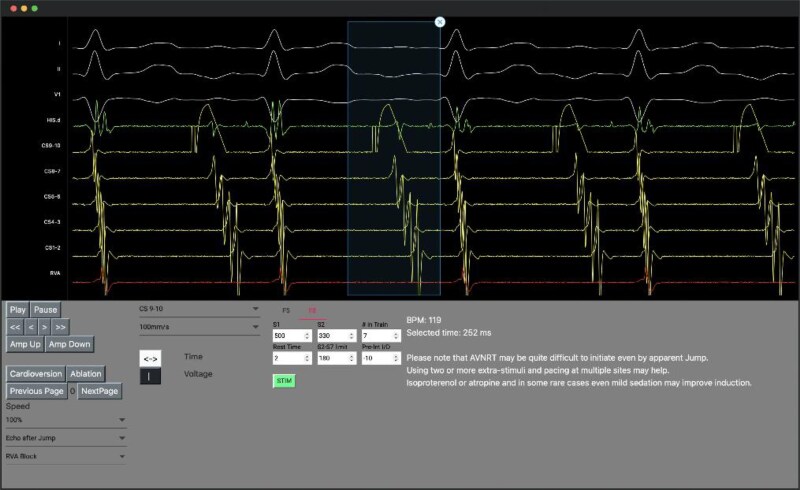
Example of pacing maneuvers on the web-based simulator EP-Simulator^TM^.

Electroanatomic mapping platforms (CARTO™ by Biosense Webster, EnSite™ by Abbott, Opal™ by Boston Scientific, Affera™ by Medtronic and Colombus™ by Microport) include simulation modes reproducing the mapping environment for practicing map construction, electrogram interpretation, and lesion validation. Browser-based simulators like EP-Simulator™ or Epicardio^TM^ focus on arrhythmia mechanisms and ablation strategies, while CIED modules address device troubleshooting and programming (*Figure 3*).^14^ These tools excel at reinforcing theoretical concepts and decision-making but cannot develop manual dexterity.

### 2.5 Virtual and augmented reality

**Figure 4:**
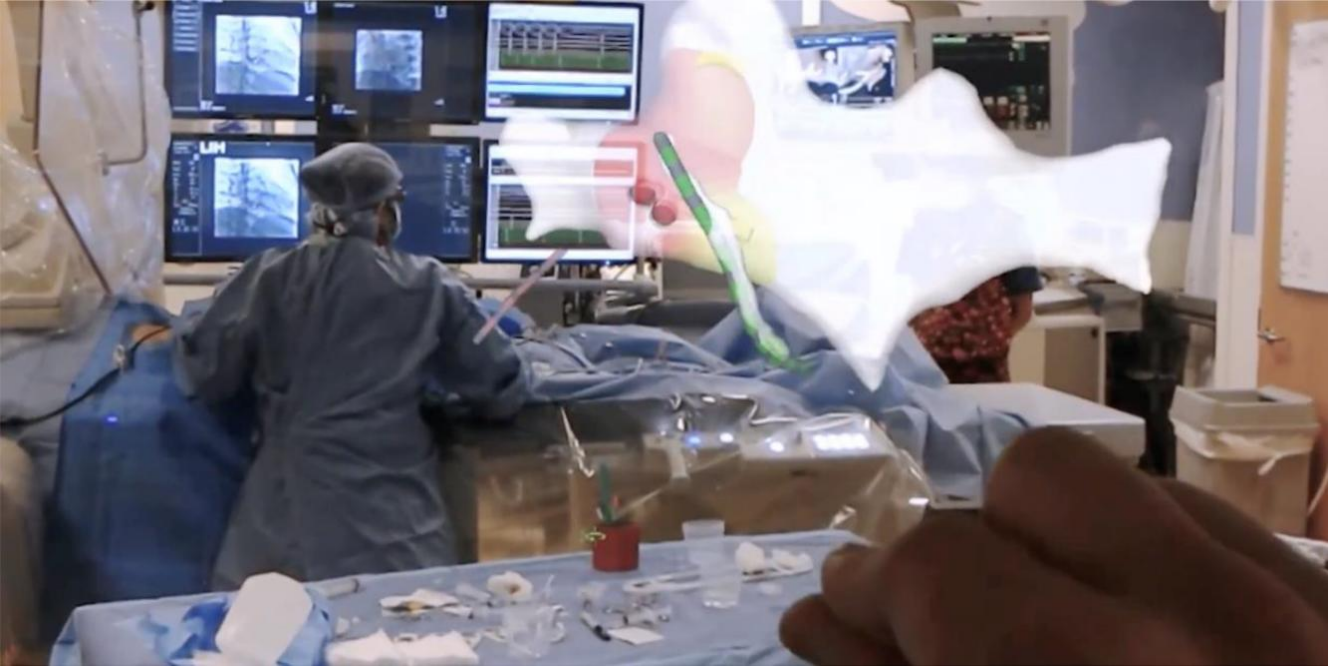
Virtual reality simulators such as CommandEP™ (Case Western Reserve University, Cleveland, OH). First-person view through an AR headset showing holographic cardiac anatomy superimposed on the real procedural environment. The visible hand illustrates the ‘mixed reality’ experience where trainees interact with virtual structures while maintaining awareness of their physical workspace.

Immersive virtual reality (VR)/AR platforms such as CommandEP™ (Case Western Reserve University, Cleveland, OH) project interactive three-dimensional anatomy using tracked controllers to enhance spatial reasoning. Heart Rhythm VR™ recently launched modules for S-ICD implantation and pericardiocentesis, with transcatheter pacemaker modules in development (*Figure 4*). Pilot studies suggest improved ablation performance in VR-trained individuals, though current limitations include absent haptic feedback and the need for curriculum validation.^15,16^

### 2.6 Integration considerations

The diversity of available simulation modalities reflects the complexity of EP and CIED procedures. Selection should align with specific learning objectives: phantoms for basic catheter skills, high-fidelity simulators for complete procedures, software for cognitive skills, and VR/AR for spatial understanding. Optimal training programmes will likely combine multiple modalities in structured progression, matching simulator complexity to trainee experience level.

## 3. Where we are, where we go: current and future use of simulation

### 3.1 Current landscape

#### 3.1.1 Academic and local initiatives

The technical capabilities described in the previous section contrast sharply with implementation. The 2024 EHRA survey on simulation training revealed that only 18% of respondents reported institutional access to EP simulators and only 20% reported formal inclusion within cardiology curricula, yet >80% of respondents rated simulation as useful or very useful for training. The survey also identified priority training areas including CSP, ventricular tachycardia (VT) ablation, and cardiac resynchronization therapy (CRT) optimization.^17^

#### 3.1.2 Industry-driven training

In practice, access is largely mediated by manufacturers, with 69% of respondents reporting simulator exposure through industry-organized training. While these initiatives can be high quality, this dependence raises concerns regarding educational neutrality and unequal access across regions. This gap supports the need for academically governed frameworks that define minimum standards and validated competency benchmarks.

#### 3.1.3 The EHRA simulation village: a neutral access model

**Figure 5:**
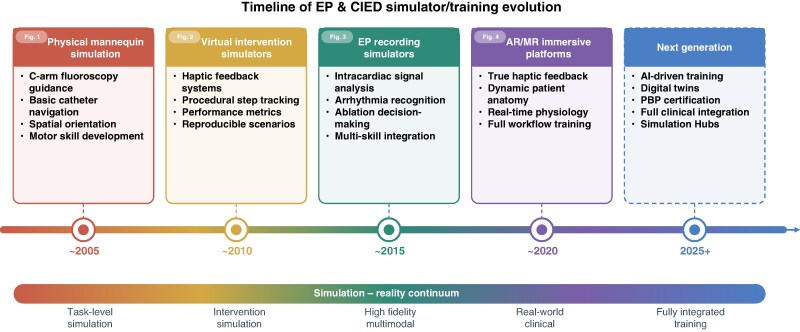
Timeline and the advancement of the different simulators.

The EHRA Simulation Village, launched in 2023, provides a supervised environment in which trainees can compare modalities and platforms under scientific society oversight (*Figure 5*). High acceptance by participants supports this model as a scalable blueprint, ideally evolving into regional hubs linked to standardized assessment and formal certification pathways.

### 3.2 Future directions

The transition from the current fragmented implementation to comprehensive integration will require structured methodological and governance frameworks. Priority areas should be guided by training needs identified in the EHRA survey, including CSP, VT ablation, and CRT.^17^

## 4. The need for protocolized evaluation: the proficiency-based progression—moving from access to excellence

### 4.1 Standards for simulation development and validation

Every EP simulator must undergo structured validation to confirm both its fidelity to reality and its educational effectiveness. Current validation methodologies range from qualitative expert evaluation to prospective randomized studies assessing skill transfer to clinical practice.^18^ However, most studies suffer from small sample sizes and lack rigorous methodology necessary to support PBP implementation, a competency-based approach in which trainees advance only after achieving predefined performance benchmarks.^19,20^

Effective simulation development must extend beyond creating high-fidelity virtual environments. The simulator should constitute a comprehensive learning platform where every procedural step is reproduced in correct sequence with appropriate devices, technical challenges match real-world complexity, and haptic and visual feedback accurately replicate clinical sensations. Performance metrics are precisely measured against predefined criteria, and formative feedback is provided both during tasks (especially for errors) and as post-procedure summative reports.^21^

Critical to PBP is quantitative performance evaluation. A small expert panel (typically three) must identify key procedural steps defining operator proficiency, then establish explicit, unambiguous metrics for each step.^22^ These metrics, including procedure duration, fluoroscopy time, contrast volume, attempt frequency, error rates, and critical errors, require benchmark values derived from expert performance. Ideally, a larger international Delphi panel validates these definitions, metrics, and benchmarks until consensus is achieved such as EHRA expert consensus statements such as for CIED implantation and CSP implantation.^23–25^

Immediate error correction, fundamental to ‘deliberate practice,’ mirrors musical training where conductors provide real-time feedback until desired quality is obtained.^26,27^ This approach prevents early-stage repetition of suboptimal techniques, with evidence showing PBP simulation training reduces recurrent errors in subsequent patient procedures.^5^

### 4.2 Building a proficiency-based training curriculum

Simulators achieve their potential only when integrated into structured curricula.^22^ A comprehensive PBP curriculum for EP/CIED training includes:

- **Baseline Knowledge Education**—Pre-simulation preparation covering relevant anatomy, procedural materials, optimal techniques, potential complications, and simulator operation. This foundation, delivered in-person or online, must be assessed against benchmarks before simulation training begins.
- **Proficiency-Driven Simulation Training**—Training duration determined by competency achievement rather than fixed timeframes. Continuous metric monitoring with immediate error correction guides trainees towards predefined proficiency levels representing optimal performance for straightforward procedures.^28^ Complex procedures may require phased progression through tutored and independent practice.^5^ Mandatory debriefing sessions, guided by metric scores, reinforce learning.^29^
- **Summative Skills Assessment**—Formal evaluation confirming that both knowledge and procedural proficiency benchmarks are met before clinical practice.

Ideally, curricula should include post-training assessment of skill transfer to patient procedures, monitoring clinical outcomes to validate training effectiveness. In CRT training, PBP demonstrated superiority over traditional simulation, where proficiency benchmarks were optional, resulting in incomplete skill development despite expert supervision.^30^ Meta-analysis of 12 medical simulation studies shows PBP training reduces procedural errors by 60%, procedure time by 15%, and increases correctly executed steps by 47% compared to conventional simulation.^18^

### 4.3 Integrating PBP into the learning journey

PBP simulation addresses multiple challenges in EP/CIED training.^9,31^ It enables an objective structured assessment of technical skills across all subspecialty aspects. Laboratory overcrowding limiting hands-on exposure becomes less critical when trainees achieve foundational proficiency through simulation. Individual variation in learning pace is accommodated through competency-based rather than time-based progression.

**Figure 6:**
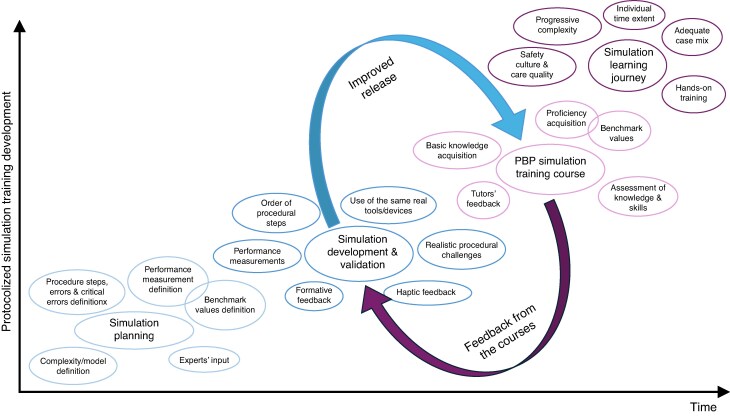
**Central Figure.** The training simulation journey from early steps through PBP and validation.

Crucially, PBP ensures systematic exposure to diverse cases with graduated complexity. Training can progress logically from basic skills (ultrasound-guided vascular access) through intermediate procedures (typical flutter ablation) to advanced techniques (complex VT/atrial fibrillation ablation), independent of clinical case availability (*Figure 6*).

## 5. The need to invent new teaching methods

### 5.1 The halstedian legacy's end

The ‘see one, do one, teach one’ model served medicine for over a century but fails modern ethical and practical standards,^32^ particularly given the volume and complexity of contemporary EP procedures within limited training timeframes.

The 2019 EHRA Young EP Ambassadors survey confirmed these systemic deficiencies: structured EP/CIED programmes absent in 51% of ESC countries, with national certification required in only 19%.^33^

### 5.2 Digital era educational demands

Beyond simulation access detailed previously, EP professionals must master digital ecosystems, including remote monitoring platforms —which increasingly provide multi-domain clinical data requiring specific training for proper interpretation—AI-assisted mapping, cybersecurity protocols, and data governance frameworks.^34^

### 5.3 Four pillars for educational transformation

**Figure 7:**
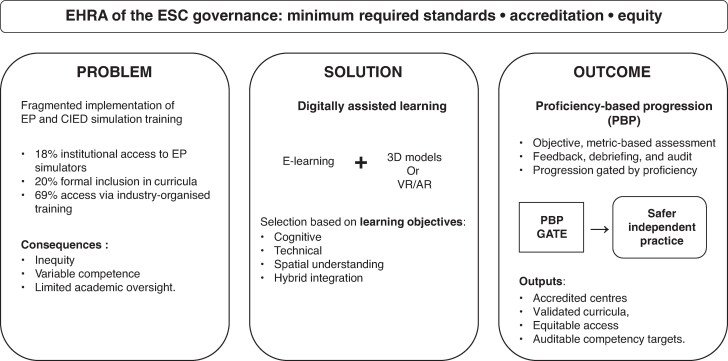
From implementation gaps to a governed, proficiency-based digital training pathway in EP and CIED.

Building on PBP principles, we propose four implementation pillars (*Figure 7*):

- **Industry-Independent Accreditation** Training centres and mentors require certification based on objective criteria: procedural volumes, case diversity, simulation resources, supervision ratios, and faculty pedagogical training. Independence from commercial influence ensures educational integrity.
- **Integrated Theoretical and Practical Curricula**
  - Theoretical foundations: delivered through online platforms, in-person sessions, ideally a combination of both modalities to optimize learning outcomes.
  - Technical skills development: Progression through simulation modalities—from task trainers through high-fidelity mannequins to VR/AR environments and 3D-printed models^35–37^
  This blended approach ensures reproducible, standardized learning experiences regardless of location.
- **Objective Competency Assessment** Progression based on validated metrics rather than time or case numbers. Proficiency checklists, performance benchmarks, and standardized evaluations ensure transparent, fair assessment aligned with PBP principles.
- **Competency-Based Certification** Academic certification independent from industry, applicable to both novice fellows and experienced physicians adopting new technologies. Certification must reflect continuous professional development, not single-point achievement.

### 5.4 Integration with existing frameworks

A distinction must be made between device-specific simulators (leadless pacemakers, electroporation platforms, mapping systems) that inherently require manufacturer involvement for development and maintenance, and generic procedural simulators (transseptal puncture, vascular access, basic catheter manipulation) that can be developed with greater academic independence.

We propose a hybrid model whereby EHRA certifies independent university-affiliated training organizations centres (‘EHRA-certified training hubs’) that meet defined and verified educational standards, as well as academic assessment and certification. These EHRA-certified university-affiliated hubs could then provide large-scale training while maintaining their academic oversight and pedagogical neutrality, and more realistically meeting the challenge of scalability. It is clear, however, that this model cannot and should not develop independently of companies, which must be involved in most phases of teaching programme design. In particular, engineers and educational staff from companies should contribute to integrating aspects that are directly related to the technological specificities of the systems covered by the training modules. The direct participation of companies is also, of course, required for the development of simulators, in order to ensure their quality, reliability, and, above all, their reproducibility of the conditions of use of the system studied in real life. This new educational model must be based on a private-public partnership between academia, scientific societies, European Union health departement and industry, with the participation of stakeholders in the scientific, technological, economic, and educational fields.

This model acknowledges industry's essential role while preserving academic governance of training standards. Such certified centres could deliver standardized, high-volume simulation training while preserving educational neutrality, academic governance, and competency assessment, under the auspices of EHRA.

These strategies align with the 2024 EHRA core curriculum^31^ and ESC Roadmap for cardiovascular education,^38^ supporting flexible, modular, digitally integrated training across Europe. Implementation requires coordinated action from scientific societies, academic institutions, and regulated industry partnerships.

## 6. Educational pathways: advantages and difficulties moving toward certification

### 6.1 Current certification framework: from theory to practice

The pedagogical innovations outlined require formal certification pathways. Cardiology training follows established principles monitoring trainee progress through Entrustable Professional Activities (EPAs), i.e. discrete professional tasks that trainees may be entrusted to perform with decreasing supervision as competence is demonstrated.^39^

EHRA's current pathway combines three elements already discussed:

- **Knowledge acquisition**: Online/onsite teaching, continuous medical education, validated by online examination (Level 1) based on predetermined syllabus
- **Skills development**: Following Objective Structured Assessment of Technical Skills (OSATS), i.e. structured checklists and global rating scales used to evaluate procedural technique and safety, progressing to procedural independence
- **Documentation**: Logbook/e-portfolio recording minimum procedure numbers, validated by senior mentors (Level 2) and EHRA certification committee

### 6.2 Integrating digital tools into certification pathways

- Integrating digital tools into certification pathways can strengthen competence-based training while improving standardization and safety. The proposed digital integration enhances this framework: **Online theoretical preparation**. Trainees complete online theoretical preparation through video-based teaching and web-based EP simulators to consolidate foundational knowledge.
- **Simulation training**. Structured simulation training provides onsite practice with objective proficiency metrics, ensuring skills acquisition is measurable rather than assumed.
- **Clinical application.** Clinical application then becomes conditional: patient procedures are undertaken only after the trainee has demonstrated predefined simulator proficiency thresholds.
- **Competency validation.** Finally, competency validation is confirmed through independence assessments supported by structured portfolios, documenting progress, case exposure, reflective learning, and supervisor sign-off to ensure readiness for autonomous practice.

### 6.3 Advantages of digitally-enhanced certification

Digitally enhanced certification is particularly well suited to EP and CIED training. Many core competencies can be assessed objectively through tracing-based questions and image-driven clinical scenarios that standardize the evaluation of knowledge and diagnostic reasoning. In parallel, technical performance can be quantified using simulator-derived metrics, provided these measures are predefined, validated, and embedded within a PBP framework. Implemented correctly, this approach supports a safer transition to independent practice by reducing avoidable early learning-curve complications and ensuring consistent baseline competencies across European centres. It may also decrease reliance on animal models by offering effective, ethically preferable training alternatives.

### 6.4 Implementation challenges

Implementing digitally enhanced certification faces several practical barriers. High-fidelity simulators are expensive and unevenly distributed, creating geographic disparities and a risk of two-tier readiness where access, rather than competence, determines progression. Recognizing that simulator costs and ongoing update requirements exceed most academic budgets, sustainable implementation requires structured academic–industry partnerships. The goal of all stakeholders being the optimal training of practitioners, we advocate for transparent collaboration wherein the industry maintains simulator currency while academic bodies ensure educational governance through EHRA certification frameworks and EHRA-certified training hubs. Ultimately, simulation costs should be recognized as a patient safety investment and funded by departments of health accordingly, rather than continuing to rely on patients as training platforms for novice operators.

Moreover, simulation fidelity remains limited for key clinical domains such as team communication, comorbidity management, and real-time decision-making under time pressure; differences between single-use clinical materials and reusable simulator components may also promote negative training transfer.

Scientific societies must navigate complex relationships, balancing investment in independent tools against collaboration with industry, with explicit governance to avoid perceived conflicts, or adopt hybrid approaches with clear boundaries.

Finally, integration requires protected educational time; otherwise, digital tools become optional add-ons rather than structured training components.

### 6.5 Pathways forward

A pragmatic pathway forward is a tiered implementation of digitally enhanced certification. A basic track should rely on widely accessible tools, like online content, standardized cognitive assessment, and low-cost task trainers, while advanced certification can incorporate high-fidelity simulation where available. Transition periods are essential to allow centres time to acquire resources and avoid widening inequities. Resource-limited institutions cannot be expected to independently acquire expensive simulation infrastructure. Therefore, several complementary strategies should be developed: regional simulation hubs serving multiple centres; EHRA-certified training organizations capable of high-volume delivery; mobile simulation units deployable at institutions or regional meetings; shared access arrangements between neighbouring centres; and fellowship grants or sponsorship programmes enabling trainees from under-resourced environments to access simulation training.

Robust quality assurance is critical, including minimum simulator specifications, validated metrics, and scheduled curriculum updates. Finally, academic institutions, scientific societies, and industry should collaborate through transparent partnerships, with clear governance to protect educational integrity while enabling innovation.

## 7. Role of scientific societies

### 7.1 Unique position and responsibilities

With certification pathways defined, scientific societies must lead implementation. Organizations like EHRA of the ESC, HRS, APHRS (Asia Pacific Heart Rhythm Society), CHRS (Chinese Heart Rhythm Society), and LAHRS (Latin American Heart Rhythm Society) and regional societies possess unique authority to set standards, certify competencies, and guide innovation while maintaining independence from commercial interests.

### 7.2 Setting educational standards

The ESC Core Curriculum and EHRA Core Curriculum already recognize simulator use early in training,^31^ providing the framework, but implementation remains fragmented.

### 7.3 Facilitating evidence generation

Societies coordinate multi-centre research identifying educational gaps and validating new methodologies. The EHRA/HRS/APHRS/LAHRS consensus on remote monitoring standardized data elements while highlighting research needs,^40^ demonstrating how societies bridge clinical practice and educational requirements. EHRA's perspectives on the ‘digital data revolution’ outline how societies can mediate between clinicians, industry, and regulators to ensure appropriate technology evaluation.^41^

### 7.4 Ensuring global equity

Scientific societies bear responsibility for reducing training disparities. Digital platforms enable participation from resource-limited settings, with online fellowships, multilingual resources, and remote modules expanding access. EHRA's webinars expansion and cross-society collaborations show how digital tools can promote inclusivity when actively prioritized.^42,43^

### 7.5 Neutral governance and stakeholder coordination

As independent entities, societies uniquely facilitate productive interactions between academia, industry, and regulatory bodies. They can:

- Establish transparent governance structures for industry collaboration
- Ensure educational content remains unbiased despite commercial support
- Validate training tools and curricula through peer review
- Advocate for regulatory recognition of simulation-based competencies

### 7.6 Implementation strategies

The EHRA survey highlights societies’ crucial role in bridging this educational gap.^17,31,32,34,44^ The EHRA Simulation Village brings one solution, but broader initiatives are needed.

To translate these responsibilities into practice, scientific societies should implement a coordinated strategy across four domains.

1. They should define certification standards by specifying minimum simulation requirements at each competency level, establishing validated proficiency metrics, and formally recognizing simulation-based training hours. Certification criteria should incorporate flexibility, and standard-setting bodies should include representation from centres of varying sizes and resource levels to ensure requirements remain realistic and inclusive.
2. Access must be expanded through regional simulation hubs serving multiple centres, complemented by mobile units and subsidized models for resource-limited institutions. As these resource-limited institutions cannot be expected to independently acquire expensive simulation infrastructure, several complementary strategies should be developed, among them: regional simulation hubs serving multiple centres; EHRA-certified regional training university-affiliated hubs organizations capable of high-volume delivery (see above); mobile simulation units deployable at institutions or regional meetings; shared access arrangements between neighbouring centres; and fellowship grants or sponsorship programmes enabling trainees from under-resourced environments to access simulation training modules in the aforementioned EHRA-certified hubs. Additionally, certification pathways should accommodate these realities through tiered requirements that do not penalize institutions lacking on-site simulators, and for these reasons, must be deployed at the European Union level. Governance frameworks must balance quality standards against accessibility.
3. Societies should foster innovation via multicentre educational technology grants and shared platforms for validated curricula and case libraries, with annual updates reflecting technological advances. The ERIS (European Research Instiute for Simulation) project could be a pillar of this strategy. Supported by EU funding, it represents an emerging initiative to establish standardized simulation infrastructure across European centres. This collaborative effort aims to develop shared curricula, validated assessment tools, and networked training facilities under EHRA coordination.
4. Finally, quality control requires accreditation of simulation centres, faculty development in digital teaching and debriefing, and periodic audits linking training outputs to educational and clinical outcomes.

## 8. Limitations and barriers

**Figure 8:**
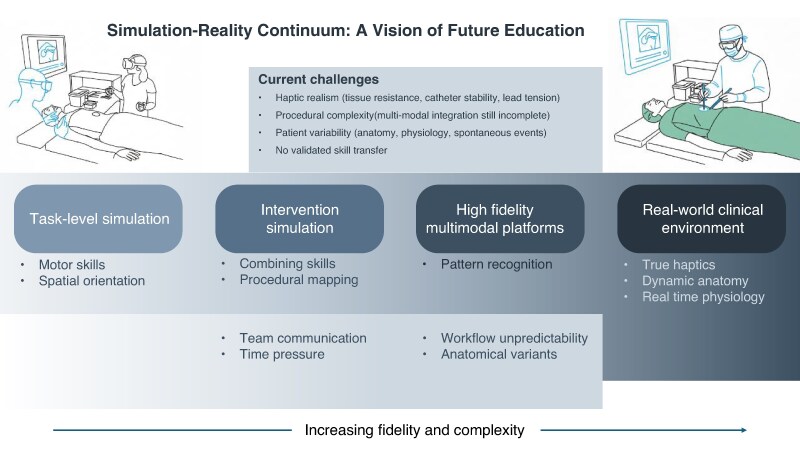
Simulation-reality continuum: a vision of future education.

Despite the progress outlined in previous sections, significant barriers remain (*Figure 8*).

### 8.1 Technical limitations

#### 8.1.1 Procedural complexity

As highlighted previously, multi-modal integration remains technically incomplete.^11,13,45,46^ Consequently, even the most advanced systems offer an approximation rather than a replication of the procedural environment.

#### 8.1.2 Haptic fidelity

Critical sensations, tissue resistance, optimal lead tensioning, and catheter stability assessment require subtleties that current technology cannot reproduce.

### 8.2 Educational barriers

#### 8.2.1 Skill transfer validation

While studies report improved performance in specific tasks, comprehensive data on patient outcomes, complication rates, and long-term skill retention are lacking.^47–49^ Equally important, simulation cannot yet replicate the contextual and interpersonal dimensions of clinical practice. Real-world procedures unfold within multidisciplinary teams under time pressure, shaped by patient-specific variability and spontaneous teaching interactions. These dynamic elements, ranging from intraoperative communication to situational judgment, remain largely absent in digital learning environments, limiting the completeness of simulation-based education.^48^

#### 8.2.2 Systemic challenges

Among the systemic challenges facing simulation-based training, resource inequality stands as a primary barrier. Resource Inequality. High-fidelity simulators require substantial investment, often exceeding €500,000 for comprehensive systems.^31,41–43,50^ High-fidelity simulation platforms represent substantial capital investments. Beyond acquisition costs, the rapid pace of EP and CIED technological evolution creates continuous need for updates: each new technique, catheter design, energy source, or device system may require costly software and hardware adaptations. Failure to maintain simulator currency results in training on outdated techniques. This economic reality largely explains why simulator development remains industry-driven; few academic institutions can independently sustain such investments, what should ideally change. Compounding this challenge is the absence of standardized simulation requirements or validated curricula. Unlike transesophageal echocardiography or interventional cardiology with established simulation frameworks, EP/CIED training lacks unified standards.^13,47,51–53^

### 8.3 Path forward despite limitations

These limitations should not discourage the advancement of simulation-based learning but rather inform its evidence-based evolution. Simulation should be viewed as an enhancement rather than a replacement for clinical experience, reinforcing procedural understanding while preserving the indispensable apprenticeship model of patient-centered training. Future integration should proceed along graduated levels of complexity, aligning the sophistication of simulation tools with specific educational objectives and learner stages. The field must now prioritize rigorous research linking simulation-based learning to measurable clinical outcomes, thereby establishing its value beyond technical proficiency alone. Parallel efforts should promote equitable access through regional collaboration, shared resource networks, and scalable, lower-cost simulation solutions. Ultimately, formal recognition of validated simulation competencies within certification frameworks will be essential to drive sustainable implementation. Universal simulator access for every fellow and every procedure, while attractive in theory, is not immediately feasible. Certification requirements must therefore be designed at the European Union level, with equity at the forefront: graduated implementation timelines, alternative pathways not requiring on-site simulators, and supported access through regional open EHRA-certified hubs, mobile units, EHRA-certified training centres, and fellowship sponsorship programmes are essential to ensure that standardization does not become exclusionary. Governance frameworks must balance the pursuit of objective, high-quality training standards against the risk of excluding centres with limited resources. The goal is to elevate and standardize training quality universally, not to concentrate influence in well-resourced institutions. Certification criteria should therefore incorporate flexibility—recognizing diverse pathways to competency—while maintaining core standards. Representation from centres of varying sizes and resource levels in standard-setting bodies will help ensure that requirements remain realistic and inclusive. In addition, accessibility to EHRA-certified training hubs must be guaranteed to all European practitioners, regardless of their gender, country, or the size of their centre. Only through coordinated academic, regulatory, and technological evolution can digital training evolve from fragmented initiatives into a fully integrated and standardized component of modern EP education.

## 9. Areas for research

### 9.1 Priority research areas

The integration of digital learning into EP/CIED education reveals critical knowledge gaps requiring systematic investigation:

**Clinical Outcome Validation**—Prospective studies must establish links between simulation-based training and patient outcomes, complication rates, procedural success, long-term results. Moving beyond surrogate markers to clinical endpoints will provide evidence necessary for regulatory recognition and institutional investment.

**Optimal Curriculum Design**—Research should determine ideal combinations of digital and traditional methods, optimal timing for simulation integration, and minimum proficiency thresholds for safe clinical practice. Comparative effectiveness studies can identify which modalities best serve specific learning objectives.

**Technology Development**—Advancing haptic feedback, complication simulation, and team-based scenarios requires continued innovation. Artificial intelligence applications for personalized learning pathways and automated performance assessment merit exploration.

**Implementation Science—**Understanding barriers to adoption, effective change management strategies, and sustainable funding models will facilitate systematic rather than opportunistic implementation across diverse healthcare systems.

**Optimal Curriculum Design**: Critically, evidence-based recommendations for minimum numbers of simulated procedures before clinical practice (e.g. transseptal punctures, vascular access, lead placements) remain to be established. Future studies should define procedure-specific thresholds validated against clinical performance benchmarks, aligned with the EHRA Core Curriculum (Trines et al., Europace 2024).

## 10. Conclusion

The advent of new digital and simulation techniques provides the perfect momentum to reconsider and reorganize teaching in EP and cardiac devices implantation, from the lecture, to the operating theatre, including the reasoned and rational use of these new tools.

The role of scientific societies is fundamental to convene stakeholders, define minimum educational standards, and ensure transparent governance of partnerships. Although challenging, this paradigm can succeed through coordinated European action despite heterogeneity in training structures and resources, by combining scalable digital learning with regional simulation hubs, validated proficiency metrics, and equitable access models that allow harmonized competency targets across centres.

## Supplementary Material

euag081_Supplementary_Data

## Data Availability

No new data were generated or analysed in support of this research.

## Abbreviations

AR: Augmented Reality
CIED: Cardiac Implantable Electronic Devices
CRT: Cardiac Resynchronization Therapy
CSP: Conduction System Pacing
EHRA: European Heart Rhythm Association
EP: Electrophysiology
HRS: Heart Rhythm Society
OSATS: Objective Structured Assessment of Technical Skills
PBP: Proficiency Based Progression
VR: Virtual Reality
VT: Ventricular Tachycardia
