# Supplementary material for: Digitally assisted learning in cardiac electrophysiology and cardiac implantable electronic devices: a Scientific Statement of the European Heart Rhythm Association of the ESC
Source: Europace. 2026 Apr 13;28(6):euag081. doi: 10.1093/europace/euag081 (PMC13303083; doi:10.1093/europace/euag081)
Supplement: euag081_Supplementary_Data [file euag081_supplementary_data.zip › Supplement figure.docx]

**Supplementary Figure:** Multi-stakeholder benefits of simulation-based training in EP/CIED procedures. Simulation-based training provides benefits across multiple stakeholder groups. Trainees gain from adaptive learning with immediate feedback. Patients benefit from enhanced safety and reduced complications. Healthcare systems see reduced radiation exposure and costs. Institutions achieve standardized assessment and reduced liability.
