# Supplementary material for: Digitally assisted learning in cardiac electrophysiology and cardiac implantable electronic devices: a Scientific Statement of the European Heart Rhythm Association of the ESC
Source: Europace. 2026 Apr 13;28(6):euag081. doi: 10.1093/europace/euag081 (PMC13303083; doi:10.1093/europace/euag081)
Supplement: euag081_Supplementary_Data [file euag081_supplementary_data.zip › Supplementary_Figure_S1.pptx]

## Slide 1
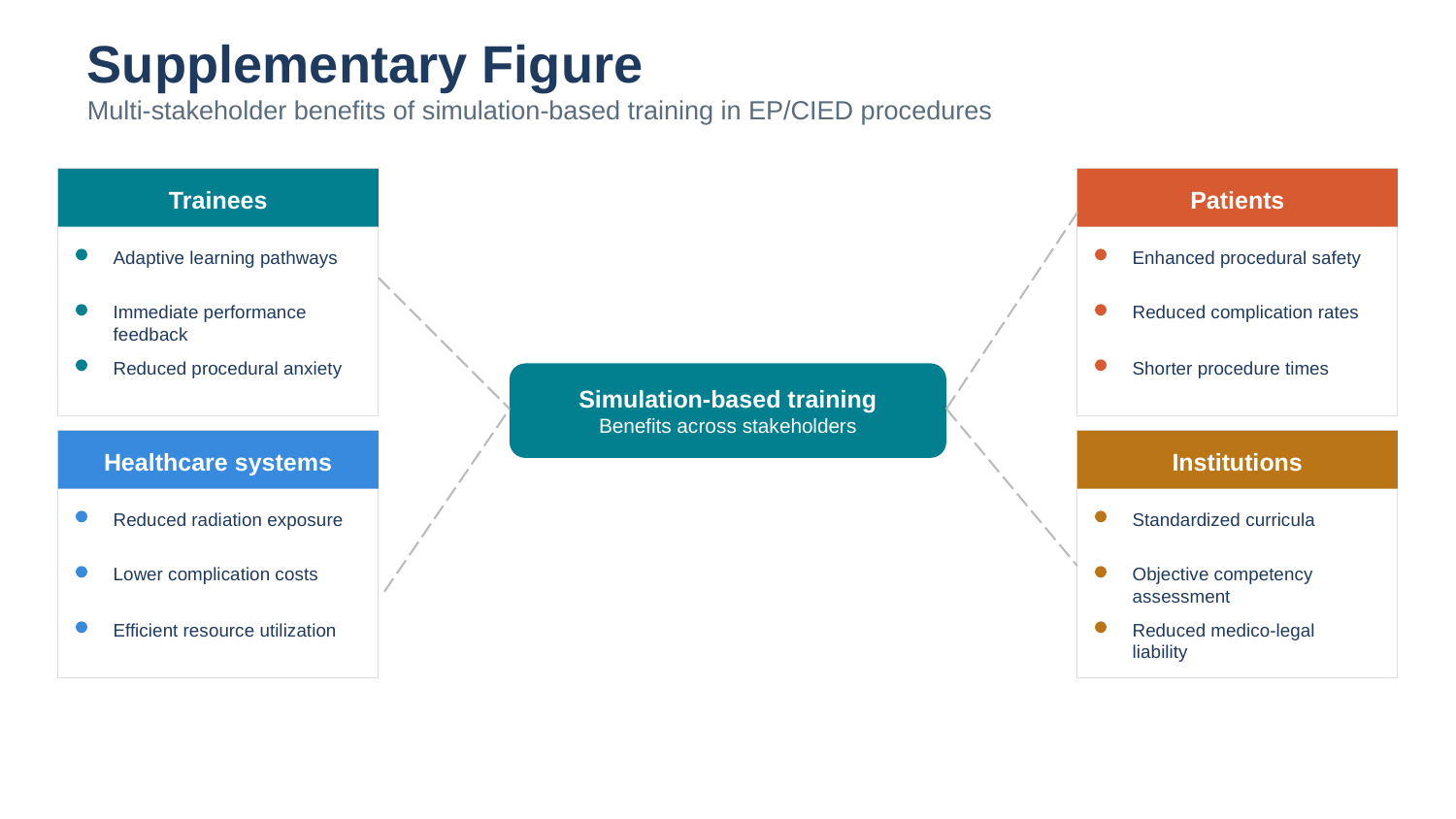

Supplementary Figure
Multi-stakeholder benefits of simulation-based training in EP/CIED procedures
Trainees
Patients
Adaptive learning pathways
Enhanced procedural safety
Immediate performance feedback
Reduced complication rates
Reduced procedural anxiety
Shorter procedure times
Simulation-based training
Benefits across stakeholders
Healthcare systems
Institutions
Reduced radiation exposure
Standardized curricula
Lower complication costs
Objective competency assessment
Efficient resource utilization
Reduced medico-legal liability
